# Analytical study of mode filtering in a duct with resistive layers

**DOI:** 10.1371/journal.pone.0339192

**Published:** 2026-01-27

**Authors:** Hani Alahmadi, Muhammad Afzal, Usman Javeed, Tayyab Nawaz

**Affiliations:** 1 Department of Mathematics, College of Science, Jouf University, Sakaka, Saudi Arabia; 2 Center for Applied Mathematics and Bioinformatics, Department of Mathematics and Natural Sciences, Gulf University for Science and Technology, Hawally, Kuwait; 3 Department of Mathematics, Capital University of Science and Technology, Islamabad, Pakistan; 4 Department of Mathematics, University of Illinois Urbana-Champaign, Champaign, Illinois, United States of America; Beni-Suef University, EGYPT

## Abstract

This paper presents an analytical mode-matching framework to examine acoustic wave propagation in a cylindrical waveguide structure featuring a central porous cavity bounded by flexible membrane discs. Unlike conventional models that consider rigid or purely absorptive boundaries, the proposed approach accounts for the dynamic response of membranes and the coupled behavior of air and porous media, enabling accurate representation of fluid–structure interactions. The acoustic field is decomposed into symmetric and anti-symmetric modal components to capture key physical phenomena such as mode conversion, energy dissipation, and complex reflection–transmission mechanisms. Continuity conditions at the interfaces are applied to determine the interaction of wave modes between subdomains, allowing the calculation of reflected, transmitted, and absorbed acoustic powers. Numerical results demonstrate strong reflection and efficient low-frequency absorption, with negligible transmission. Parametric analysis reveals that increasing the length of the porous cavity enhances sound attenuation by intensifying dissipative effects. These findings highlight the effectiveness of the multilayered configuration as a frequency-selective acoustic filter, offering a tunable and practical solution for silencers and noise control in engineering structures.

## 1 Introduction

The control of acoustic wave propagation in ducted structures is essential for a wide range of applications, including noise reduction in ventilation systems, HVAC, exhaust mufflers, biomedical devices, and acoustic metamaterial design. Modeling wave propagation in ducts with porous and flexible boundaries is of great significance in the context of acoustic filters, silencers, and energy dissipation devices. The use of porous materials within cavities enables energy absorption, while flexible boundaries such as membranes allow for interaction between the structural and acoustic fields. Analytical modeling of such configurations requires both accurate physical models for wave propagation in complex media and mathematically tractable solution techniques.

A range of studies have advanced the understanding and control of acoustic attenuation in duct systems and structural enclosures. Selamet and Radavich [1] analyzed the acoustic behavior of concentric expansion chambers and highlighted how chamber length affects attenuation through a combination of theoretical, numerical, and experimental approaches. Lee and Kim [2] explored the design of muffler configurations using topology optimization to enhance their acoustic efficiency. Maxit et al. [3] developed a modeling technique based on patch transfer functions to account for micro-perforated panels in complex vibro-acoustic systems. Expanding on the interaction between structural features and acoustic fields, Yu et al. [4] examined systems incorporating apertures and perforated components, and later investigated how mixed flexible structures with openings influence sound transmission [5]. These contributions collectively offer important methodologies and insights for improving noise control in engineered systems. On the other hand, the flexible structures containing perforations backed by air cavities, offer a practical solution in settings where traditional porous materials are unsuitable. The performance of such microperforated panels (MPPs) is highly dependent on cavity configuration and structural design, and have been explored using both theoretical and experimental approaches. Zhang and Gu [6] developed a theoretical framework for double-layered systems microperforated panel (MPP), highlighting how layering and spacing affect acoustic energy dissipation. Toyoda and Takahashi [7] further examined sound transmission behavior in MPP configurations with subdivided cavities, demonstrating the sensitivity of transmission loss to internal geometry—an aspect that benefits from modeling techniques such as the mode matching method and transfer matrix analysis. Complementing these studies, Hillereau et al. [8] evaluated single-layer liners with porous honeycomb structures and showed that liner design significantly influences attenuation, especially at low to mid frequencies. Peiwei et al. [9] investigated the modeling of silencers in trifurcated waveguide structures using different porous materials. Their results highlighted how geometric branching combined with porosity can significantly enhance acoustic attenuation in duct systems. Yairi et al. [10] emphasized the role of back cavity geometry in the absorption efficiency of MPPs, revealing opportunities for design enhancement. Building on this concept, Sum et al. [11] proposed an arrangement of multiple MPP subabsorbers in parallel to control duct-borne noise, offering a modular design strategy for improving broadband attenuation. Recently, Li et al. [12] introduced a refined analytical framework for circular microperforated membranes (MPMs), in which the velocity at the perforation boundary is treated as continuous with the membrane motion. Unlike classical models that assume stationary perforation walls, their approach accounts for the membrane’s vibration, leading to a position-dependent impedance model. Recently, Afsar et al. [13] investigated noise control in building acoustics using ducts with flexible absorbent boundaries. Their study demonstrated the potential of membrane porous configurations for enhancing low-frequency attenuation in practical applications.

Despite significant progress in acoustic modeling, the integration of porous materials into fluid–structure interaction systems—such as ducts incorporating flexible membranes—remains a complex and relatively underexplored domain. This study investigates a cylindrical waveguide setup featuring a porous central cavity coupled with flexible membrane discs at its ends. The acoustic interaction with both reactive elements (membranes) and dissipative components (porous materials) is examined through a symmetric–anti-symmetric mode-matching technique (MMT). The MMT framework involves expanding the acoustic and structural fields within each sub-domain using appropriate eigenfunctions (modes). These modal representations are then coupled across interfaces by enforcing physical continuity conditions, such as pressure and normal velocity matching. This technique has proven effective for handling discontinuities, flexible boundaries, and layered media in waveguide problems. Earlier work by Selamet and Radavich [1] demonstrated the utility of modal decomposition combined with boundary matching, emphasizing the role of geometric extensions in improving silencer performance. Nennig et al. [14] extended the method to include poroelastic materials in the presence of mean flow, enabling detailed modeling of realistic dissipative silencers. Kirby [15] offered a comparative study of analytical mode matching and numerical methods, showing their respective strengths in predicting transmission loss under flow conditions and for complex geometries. When structural flexibility is introduced, as in systems with membranes, the governing equations become more intricate due to coupled fluid–structure interactions. Lawrie [16] addressed such challenges by deriving analytical solutions for three-dimensional rectangular ducts with porous linings and compliant walls. Later, Lawrie and Afzal [17]introduced an extension of the mode-matching method that incorporates a Galerkin-based formulation to account for the dynamics of membrane discontinuities. This Galerkin approach has since been adopted in subsequent works [18–22] to model the localized response at membrane interfaces more effectively.

This study develops an analytical mode-matching framework to investigate acoustic wave propagation in a cylindrical waveguide system composed of both dissipative and reactive elements. The setup consists of a central porous region flanked by two symmetrically placed membrane discs. In contrast to conventional models that assume rigid or purely absorptive boundaries, this work incorporates flexible membranes with complex dynamic behavior, modeled through a Galerkin-based approach. This enables a detailed representation of fluid–structure interactions at the interfaces. A key aspect of the method is the combined treatment of symmetric and anti-symmetric modal components, allowing the acoustic field to be decomposed into analytically manageable subproblems. The porous segment functions as a spatially distributed dissipative layer, while the membrane boundaries contribute reactive impedance. By synthesizing modal solutions across these regions, the model captures essential wave phenomena such as modal conversion, enhanced dissipation, and coupled reflection–transmission behavior at composite boundaries. This integrated modeling strategy provides a refined understanding of sound propagation in multi-cavity systems involving both reactive and absorptive features—an area that has received limited attention in existing mode-matching literature. Despite valuable progress in the analytical and numerical modelling of duct acoustics and fluid–structure interaction, several important gaps remain in the literature that motivate the present study and future investigation. First, many existing analyses focus on rigid or simple impedance boundaries and therefore do not capture the full two-way coupling between flexible structural elements and dissipative porous cores; see e.g. [14,15,17]. Second, the porous layers are commonly represented by empirical, frequency-limited models (e.g. Delany–Bazley [23]) whose accuracy deteriorates at very low frequencies or for complex microstructures [24]. Third, mean flow, non-axisymmetric modes, and nonlinear fluid–structure effects are often neglected in analytic treatments, limiting applicability to realistic engineering conditions (e.g. ducts with grazing or through-flow). Fourth, few studies provide systematic experimental validation or uncertainty quantification, which reduces confidence when transferring analytical predictions to design practice. Finally, optimization and control perspectives such as parameter optimization or active tuning of membrane-backed cavities — have been explored only sparsely in the context of coupled porous–membrane systems.

The article is structured as follows: Sect 2 presents the governing differential equations for the coupled system. Sect 3 addresses wave propagation through a porous cavity with compliant boundary conditions. Sect 4 extends the formulation to symmetric porous-soft configurations. Numerical results and parameter studies are discussed in Sect 5, with concluding remarks summarized in Sect 6.

## 2 Problem formulation

We consider acoustic wave propagation in a cylindrical waveguide featuring a thin, flexible membrane mounted at the inlet interface located at *z* = −2*L*. This membrane acts as a structural discontinuity where incident acoustic waves interact with the elastic boundary. The system is partitioned into three distinct axial regions:

- **Region I** (*z* < –2*L*): Contains the incident acoustic wave characterized by fluid potential $\phi_{1}$. This region represents the upstream waveguide segment where waves impinge on the membrane.
- **Region II** (–2*L* < *z* < –*L*): An intermediate air cavity bounded between the flexible membrane and the porous layer. The acoustic field in this region is represented by fluid potential $\phi_{2}$.
- **Region III** (–*L* < *z* < 0): This region contains a porous material backed by a rigid termination, with the acoustic potential denoted by $\phi_{3}$.

**Fig 1:**
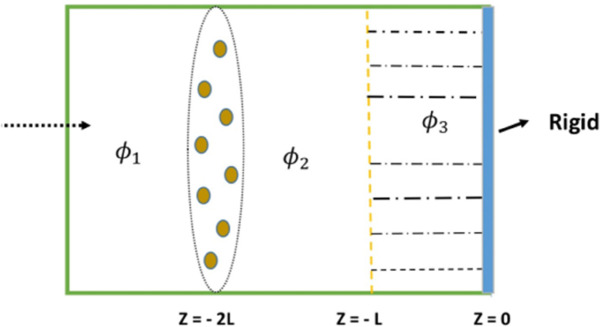
Schematic of the layered acoustic waveguide system with membrane and porous backing.

The internal medium in all regions consists of a compressible fluid with sound speed *c* and density $\rho_{0}$. Fig 1 illustrates the schematic layout of the layered waveguide system, including the membrane, air gap, and porous segment.

The governing equations employed in this study are derived from standard formulations in acoustics and structural dynamics. Specifically, the acoustic wave propagation in fluid regions is described by the Helmholtz equation [25], while wave motion in porous materials follows the modified Helmholtz-type equation incorporating dissipative effects [23,26]. The transverse vibration of the membrane is governed by the classical membrane vibration model [27].

The acoustic field in Regions I and II is governed by the Helmholtz equation [25] :

$$(\nabla^{2}+k^{2})\phi_{j}(r,z)=0,\;j=1,2,$$ (1)

where $\nabla^{2}$ is the Laplacian operator in cylindrical coordinates and k=ω/c is the acoustic wavenumber. Due to the axisymmetric nature of the problem, the travelling wave formulation modes can be expressed in terms of the zeroth-order Bessel function:

$$\left\{J_{0}(\tau_{n}r)e^{\pm i\eta_{n}z},\eta_{n}=\sqrt{k^{2}-\tau_{n}^{2}};n=0,1,2,\cdots \right\},$$ (2)

where $\tau_{n}$ are the radial eigenvalues corresponding to rigid boundary conditions at *r* = *a*. In Region III, which contains a porous material, the wave propagation is described by a modified Helmholtz equation [23,26]:

$$(\nabla^{2}+\Gamma^{2})\phi_{3}(r,z)=0,$$ (3)

where Γ is a complex parameter incorporating the dissipative effects of the porous medium. It is defined as [23,26]

$$\Gamma :=1+ia_{1}\xi^{a_{2}}+a_{3}\xi^{a_{4}},$$ (4)

with the dimensionless frequency ξ=fρ/σ and flow resistivity modeled as $\sigma =A_{1}\rho^{A_{2}}$. The complex equivalent density is given by [23,26]

$$\tau :=\Gamma \left[1+a_{5}\xi^{a_{6}}-ia_{7}\xi^{a_{8}}\right],$$ (5)

where *a*_*j*_ (for j=1,…,8) and $A_{1},A_{2}$ are empirical parameters characterizing the porous material. the coefficients $a_{1},\ldots ,a_{8}$ are empirical constants obtained from the Delany–Bazley [23] model and determine the frequency dependence of the complex wave number and effective density in porous materials. Specifically, *a*_1_–*a*_4_ control the functional dependence of the complex propagation constant Γ, while *a*_5_–*a*_8_ describe corrections associated with the effective density *τ*. The parameters *A*_1_ and *A*_2_ are scaling coefficients that relate the flow resistivity *σ* to the ambient density *ρ* via $\sigma =A_{1}\rho^{A_{2}}$. These values are material dependent and are obtained from experimental characterizations available in the literature (see [24–28]).

The traveling wave formulation modes in this region takes the form

$$\left\{J_{0}(\tau_{n}r)e^{\pm is_{n}z},s_{n}=\sqrt{\Gamma^{2}-\tau_{n}^{2}};n=0,1,2,\cdots \right\}.$$ (6)

To analyze the scattering and attenuation behavior, a boundary value problem is formulated in terms of the acoustic potential ϕ, incorporating appropriate interface and wall conditions. The problem is further non-dimensionalized to facilitate numerical implementation and parameter analysis. The dynamics of the flexible membrane located at *z* = −2*L* are described by the axisymmetric membrane vibration equation [27]:

$$\nabla^{2}U+\frac{\omega^{2}}{c_{m}^{2}}U=\frac{1}{T}{\left[p\right]}_{-}^{+},$$ (7)

where *U*(*r*) is the transverse membrane displacement, $c_{m}=\sqrt{T/\rho_{m}}$ is the wave speed on the membrane, *T* is the membrane tension, and $\rho_{m}$ is its mass per unit area. The term ${\left[p\right]}_{-}^{+}=p^{+}$ − *p*^−^ represents the pressure jump across the membrane interface. The membrane is clamped at its circular edge and satisfies the zero-displacement boundary condition:

U(r=a)=0,at z=−2L. (8)

The problem is formulated in cylindrical coordinates (r,θ,z) under axisymmetric assumptions, which eliminate θ-dependence and enable analytical modal decomposition of the acoustic and structural fields.

## 3 Galerkin formulation for axisymmetric membrane

Within the Galerkin formulation, the displacement of the membrane disc located at the interface is represented as a series expansion in orthogonal basis functions. This procedure is consistent with the methodology outlined in [17]. The problem is formulated in cylindrical coordinates (r,θ,z), assuming axisymmetry, which eliminates any dependence on the angular coordinate θ and allows for analytical modal decomposition of the acoustic and structural domains. At the membrane position *z* = −2*L*, the displacement simplifies to a function of the radial coordinate only, denoted by *U*(*r*). The governing equation for *U*(*r*) takes the form of an inhomogeneous Bessel-type differential equation:

$$\frac{1}{r}\frac{d}{dr}\left(r\frac{dU}{dr}\right)+\frac{\omega^{2}}{c_{m}^{2}}U=\frac{1}{T}{\left[p\right]}_{-}^{+}(r),\;0<r<a.$$ (9)

The membrane is clamped at the outer edge:

U(a)=0. (10)

We seek an approximate solution using the Galerkin method by expressing the displacement as a series of orthogonal eigenfunctions that satisfy the homogeneous boundary condition:

$$U(r)=\sum_{m=0}^{\infty }w_{m}J_{0}\left(\lambda_{m}r\right),$$ (11)

where $\lambda_{m}$ is the *m*-th root of *J*_0_, i.e., $J_{0}(\lambda_{m})=0$, ensuring $J_{0}\left(\frac{\lambda_{m}a}{a}\right)=0$. Substituting Eq (11) into Eq (9), and applying Galerkin projection by multiplying both sides with $J_{0}\left(\lambda_{n}r\right)$ and integrating over the domain r∈[0,a], we obtain:

$$\sum_{m=0}^{\infty }w_{m}\left(\left(\frac{\omega^{2}}{c_{m}^{2}}-{\left(\lambda_{m}\right)}^{2}\right)\int_{0}^{a}J_{0}\left(\lambda_{m}r\right)J_{0}\left(\lambda_{n}r\right)r\;dr\right)=\frac{1}{T}\int_{0}^{a}{\left[p\right]}_{-}^{+}(r)J_{0}\left(\lambda_{n}r\right)r\;dr.$$ (12)

Due to the orthogonality of Bessel functions, the integral on the left-hand side simplifies using:

$$\int_{0}^{a}J_{0}\left(\lambda_{m}r\right)J_{0}\left(\lambda_{n}r\right)r\;dr=\left\{ \begin{array}{cc} 0, & m\neq n,\\ \frac{a^{2}}{2}[J_{1}(\lambda_{m}){]}^{2}, & m=n. \end{array}\right.$$ (13)

Thus, the modal coefficients *w*_*m*_ are given by:

$$w_{m}=\frac{2}{Ta^{2}[J_{1}(\lambda_{m}){]}^{2}}\cdot \frac{\int_{0}^{a}{\left[p\right]}_{-}^{+}(r)J_{0}\left(\lambda_{m}r\right)r\;dr}{\left(\frac{\omega^{2}}{c_{m}^{2}}-{\left(\lambda_{m}\right)}^{2}\right)}.$$ (14)

Therefore, the membrane displacement at *z* = −2*L* is expressed as:

$$U(r)=\sum_{m=0}^{\infty }\left(\frac{2}{Ta^{2}[J_{1}(\lambda_{m}){]}^{2}}\cdot \frac{\int_{0}^{a}{\left[p\right]}_{-}^{+}(r)J_{0}\left(\lambda_{m}r\right)r\;dr}{\left(\frac{\omega^{2}}{c_{m}^{2}}-{\left(\lambda_{m}\right)}^{2}\right)}\right)J_{0}\left(\lambda_{m}r\right).$$ (15)

The duct wall is rigid, leading to an axisymmetric system (with axisymmetric wave forcing assumed), hence the θ coordinate is omitted.

## 4 Mode matching formulation

In the eigenfunction expansion method, the unknown modal amplitudes are determined using mode-matching techniques. Before applying this method, the inherent structural symmetry is exploited to decompose the problem into two distinct cases: symmetric and anti-symmetric configurations. These will be explored in subsequent subsections.

### 4.1 Symmetric acoustic modes for a rigid interface setup

Consider the case where the membrane disc located at *z* = 0 behaves as a rigid interface. This symmetry condition implies $D_{n}=C_{n}$, and the superscript *s* is used to denote variables in the symmetric configuration. The unknown amplitudes in this case are denoted $A_{n}^{s}$, $B_{n}^{s}$, $C_{n}^{s}$, and $D_{n}^{s}$. The eigenfunction expansions for each region take the form:

$$\phi_{1}^{s}(r,z)=e^{ik(z+2L)}+\sum_{n=0}^{\infty }A_{n}^{s}J_{0}(\tau_{n}r)e^{-i\eta_{n}(z+2L)},$$ (16)

$$\phi_{2}^{s}(r,z)=\sum_{n=0}^{\infty }\left[B_{n}^{s}e^{i\eta_{n}(z+L)}+C_{n}^{s}e^{-i\eta_{n}(z+L)}\right]J_{0}(\tau_{n}r),$$ (17)

$$\phi_{3}^{s}(r,z)=2\sum_{n=0}^{\infty }D_{n}^{s}\cos(s_{n}z)J_{0}(\tau_{n}r).$$ (18)

Here, $\eta_{n}=\sqrt{k^{2}-\tau_{n}^{2}}$ are the axial mode wavenumbers, and the eigenvalues $\tau_{n}$ satisfy $J_{0}^{\prime }(\tau_{n}a)=0$. The eigenfunctions $J_{0}(\tau_{n}r)$ form an orthogonal basis over the radial domain with respect to the weight function *r*, satisfying:

$$\int_{0}^{a}J_{0}(\tau_{n}r)J_{0}(\tau_{m}r)r\;dr=\delta_{mn}X_{m},\;\text{where}\;X_{m}=\int_{0}^{a}J_{0}^{2}(\tau_{n}r)r\;dr.$$ (19)

At the membrane location *z* = −2*L*, the equation of motion becomes

$$(\nabla^{2}+k_{p}^{2})U^{s}(r)=\alpha_{p}\left(\phi_{2}^{s}-\phi_{1}^{s}\right),$$ (20)

subject to the fixed boundary condition at the radial edge:

$$U^{s}(a)=0.$$ (21)

Assuming the membrane displacement can be represented as:

$$U^{s}(r)=\sum_{n=0}^{\infty }w_{n}^{s}J_{0}(\lambda_{n}r),$$ (22)

where $\lambda_{n}$ are roots of $J_{0}(\lambda_{n}a)=0$, and the basis functions satisfy:

$$\int_{0}^{a}J_{0}(\lambda_{n}r)J_{0}(\lambda_{m}r)r\;dr=\delta_{mn}I_{m},\;\text{where}\;I_{m}=\frac{a^{2}}{2}[J_{1}(\lambda_{m}a){]}^{2}.$$ (23)

Substituting Eqs (16), (17), and (22) into the equation of motion (20), the coefficients $w_{m}^{s}$ are determined as:

$$w_{m}^{s}=\frac{\alpha_{p}}{(k_{p}^{2}-\lambda_{m}^{2})I_{m}}\left[\sum_{n=0}^{\infty }\left(B_{n}^{s}e^{-i\eta_{n}L}+C_{n}^{s}e^{i\eta_{n}L}-A_{n}^{s}\right)R_{mn}-\delta_{m0}\right],$$ (24)

where *R*_*mn*_ are projection coefficients from modal integration. The velocity continuity condition at *z* = −*L* yields:

$$\frac{\partial \phi_{2}^{s}}{\partial z}=\frac{\partial \phi_{3}^{s}}{\partial z}.$$ (25)

Substituting Eqs (17) and (18) into (25) gives:

$$B_{m}^{s}-C_{m}^{s}=-\frac{2s_{m}}{i\eta_{m}X_{m}}D_{m}^{s}\sin(s_{m}L).$$ (26)

The pressure continuity condition at *z* = −*L* is:

$$\phi_{2}^{s}(r,-L)=\beta \phi_{3}^{s}(r,-L),$$ (27)

which upon substitution yields:

$$B_{m}^{s}+C_{m}^{s}=2\beta D_{m}^{s}\cos(s_{m}L).$$ (28)

Solving Eqs (26) and (28) simultaneously gives expressions for $B_{m}^{s}$ and $C_{m}^{s}$:

$$B_{m}^{s}=\left[\beta \cos(s_{m}L)+\frac{is_{m}}{\eta_{m}X_{m}}\sin(s_{m}L)\right]D_{m}^{s},$$ (29)

$$C_{m}^{s}=\left[\beta \cos(s_{m}L)-\frac{is_{m}}{\eta_{m}X_{m}}\sin(s_{m}L)\right]D_{m}^{s}.$$ (30)

The velocity continuity at *z* = −2*L* between the fluid and the membrane is:

$$\frac{\partial \phi_{1}^{s}}{\partial z}=U^{s}(r),$$ (31)

leading to the expression for $A_{m}^{s}$:

$$A_{m}^{s}=\frac{k\delta_{m0}}{\eta_{m}}+\frac{i}{\eta_{m}X_{m}}\sum_{n=0}^{\infty }w_{n}^{s}R_{mn}.$$ (32)

At the same interface *z* = −2*L*, the velocity continuity condition in Region II gives:

$$\frac{\partial \phi_{2}^{s}}{\partial z}=U^{s}(r),$$ (33)

which upon substitution results in:

$$B_{m}^{s}e^{-i\eta_{m}L}-C_{m}^{s}e^{i\eta_{m}L}=-\frac{i}{\eta_{m}X_{m}}\sum_{n=0}^{\infty }w_{n}^{s}R_{mn}.$$ (34)

The unknown coefficients $B_{n}^{s}$, $C_{n}^{s}$, and $w_{n}^{s}$ can be determined by truncating the modal expansions and solving the resulting system of equations numerically.

### 4.2 Anti-symmetric acoustic modes for a rigid interface setup

**Fig 2:**
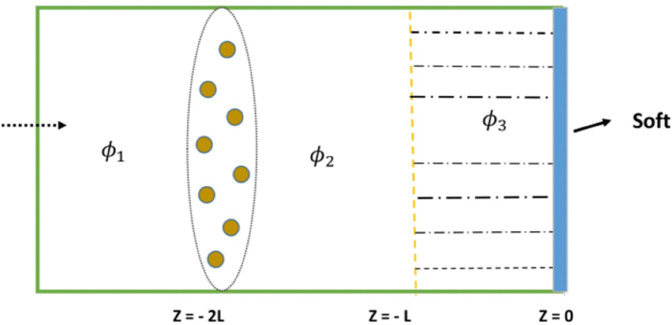
Physical configuration of waveguide.

In the anti-symmetric configuration, the eigenfunction expansion fundamentally differs from the symmetric case due to the behavior of eigenfunctions under spatial transformations such as reflection. In this case, the solution is constructed using eigenfunctions that satisfy anti-symmetric boundary conditions. These are typically odd functions, and the expansion coefficients are determined using orthogonality relations. The physical configuration of the waveguide system with a soft backing is illustrated in Fig 2.

Assuming the disc at *z* = 0 is rigid, an anti-symmetric configuration can be modeled by imposing the condition $B_{n}=-C_{n}$. In this setting, we introduce variables with the superscript a to denote anti-symmetric mode amplitudes $A_{n}^{a},B_{n}^{a},C_{n}^{a},$ and $D_{n}^{a}$. The corresponding eigenfunction expansions are given by:

$$\phi_{1}^{a}(r,z)=e^{ik(z+2L)}+\sum_{n=0}^{\infty }A_{n}^{a}J_{0}(\tau_{n}r)e^{-i\eta_{n}(z+2L)},$$ (35)

$$\phi_{2}^{a}(r,z)=\sum_{n=0}^{\infty }\left[B_{n}^{a}e^{i\eta_{n}(z+L)}+C_{n}^{a}e^{-i\eta_{n}(z+L)}\right]J_{0}(\tau_{n}r),$$ (36)

$$\phi_{3}^{a}(r,z)=2i\sum_{n=0}^{\infty }D_{n}^{a}\sin(s_{n}z)J_{0}(\tau_{n}r),$$ (37)

where $\eta_{n}=\sqrt{k^{2}-\tau_{n}^{2}}$ and $s_{n}=\sqrt{\Gamma^{2}-\tau_{n}^{2}}$, for n=0,1,2,…. At *z* = −2*L*, the membrane displacement satisfies the governing equation:

$$(\nabla^{2}+k_{p}^{2})U^{a}(r)=\alpha_{p}(\phi_{2}^{a}-\phi_{1}^{a}),$$ (38)

subject to the fixed-edge boundary condition:

$$U^{a}(a)=0.$$ (39)

Assuming a modal expansion of the form:

$$U^{a}(r)=\sum_{n=0}^{\infty }w_{n}^{a}J_{0}(\lambda_{n}r),$$ (40)

where $\lambda_{n}$ are the roots of $J_{0}(\lambda_{n}a)=0$, substituting Eqs (35), (36), and (40) into Eq (38) yields:

$$w_{m}^{a}=\frac{\alpha_{p}}{(k_{p}^{2}-\lambda_{m}^{2})I_{m}}\left[\sum_{n=0}^{\infty }\left(B_{n}^{a}e^{-i\eta_{n}L}+C_{n}^{a}e^{i\eta_{n}L}-A_{n}^{a}\right)R_{mn}-\delta_{m0}\right].$$ (41)

Continuity of velocity at *z* = −*L* gives:

$$\frac{\partial \phi_{2}^{a}}{\partial z}=\frac{\partial \phi_{3}^{a}}{\partial z}.$$ (42)

Substituting Eqs (36) and (37) into Eq (42) and applying orthogonality results in:

$$B_{m}^{a}-C_{m}^{a}=\frac{2s_{m}}{\eta_{m}X_{m}}D_{m}^{a}\cos(s_{m}L).$$ (43)

Similarly, pressure continuity at *z* = −*L* implies:

$$\phi_{2}^{a}(r,-L)=\beta \phi_{3}^{a}(r,-L),$$ (44)

which gives:

$$B_{m}^{a}+C_{m}^{a}=2i\beta D_{m}^{a}\sin(s_{m}L).$$ (45)

Solving Eqs (43) and (45) yields:

$$B_{m}^{a}=\left[\frac{s_{m}}{\eta_{m}X_{m}}\cos(s_{m}L)+i\beta \sin(s_{m}L)\right]D_{m}^{a},$$ (46)

$$C_{m}^{a}=\left[i\beta \sin(s_{m}L)-\frac{s_{m}}{\eta_{m}X_{m}}\cos(s_{m}L)\right]D_{m}^{a}.$$ (47)

The velocity continuity condition at *z* = −2*L* between regions 1 and the membrane gives:

$$\frac{\partial \phi_{1}^{a}}{\partial z}=U^{a}(r),$$ (48)

which leads to:

$$A_{m}^{a}=\frac{k\delta_{m0}}{\eta_{m}}+\frac{i}{\eta_{m}X_{m}}\sum_{n=0}^{\infty }w_{n}^{a}R_{mn}.$$ (49)

Similarly, continuity of velocity between the membrane and region 2 at *z* = −2*L* gives:

$$\frac{\partial \phi_{2}^{a}}{\partial z}=U^{a}(r),$$ (50)

yielding:

$$B_{m}^{a}e^{-i\eta_{m}L}-C_{m}^{a}e^{i\eta_{m}L}=\frac{-i}{\eta_{m}X_{m}}\sum_{n=0}^{\infty }w_{n}^{a}R_{mn}.$$ (51)

## 5 Numerical results and discussions

The problem is examined through a physical interpretation supported by numerical experiments. This is achieved by employing a truncated solution approach and assigning specific numerical values to the involved parameters. The system is truncated to *N* + 1 equations by considering the parameter indices m=n=0,1,2,…,N, where *N* denotes the truncation parameter. The internal fluid is assumed to be air, with a density of $\rho =1.2043\;{\text{kg/m}}^{3}$ and a sound speed of c=343.5m/s. The membrane is characterized by a mass density of $\rho_{m}=0.1715\;{\text{kg/m}}^{2}$ and a tension of T=350N/m. The parameters for porous materials—such as A-Glass, E-Glass, or Steel Wool—can be adopted based on experimental data provided in [28], where Kirby and Cummings present coefficient values for a variety of commonly used porous media. The advantages and limitations of the Delany and Bazley model [23] are extensively discussed by Allard and Champoux [24], as well as by Kirby and Cummings [28]. Although the Delany–Bazley formulation becomes less reliable at low frequencies, it offers a practical and convenient approximation well-suited for mode-matching analysis. For A-Glass, the empirical coefficients are given as: $a_{1}=0.2251,a_{2}=-0.5827,a_{3}=0.1443,a_{4}=-0.7088,a_{5}=0.0924,a_{6}=-0.7177,a_{7}=0.1457,a_{8}=-0.5951,$ $A_{1}=1.857,A_{2}=1.687,$ which are substituted into Eqs (4) and (5) to obtain the numerical values of Γ and τ. The dimensional parameters used in the numerical analysis include a cavity height of $\bar{a}=0.5\;m$, a chamber length of L=0.3m, and an operating frequency of f=550Hz.

**Fig 3:**
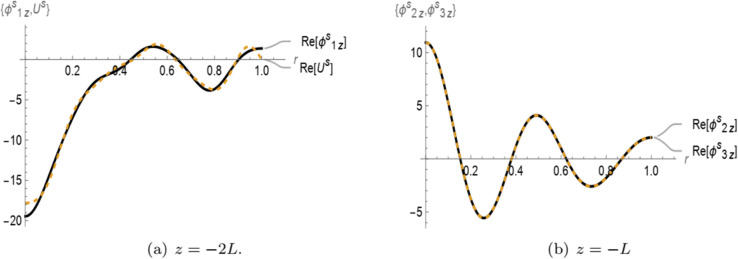
For symmetric case: The real parts of velocities and membrane displacement at interfaces z=−2L and –L.

**Fig 4:**
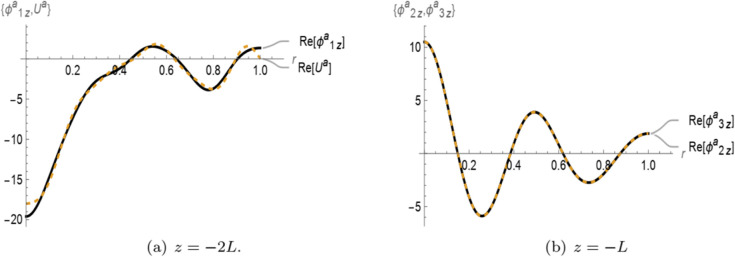
For anti-symmetric case: The real parts of velocities and membrane displacement at interfaces z=−2L and –L.

In Figs 3 and 4, the real parts of the normal velocities and membrane displacement are presented for the symmetric and anti-symmetric cases, respectively, as functions of the radial coordinate *r* at the interfaces *z* = −2*L* and *z* = −*L*. The solutions are computed using a truncation parameter of *N* = 15. In Fig 3(a), the real part of the symmetric membrane displacement *U*_1_ exhibits good agreement with the real part of the symmetric normal velocity $\phi_{1z}(r,z)$ at *z* = −2*L*. Similarly, in Fig 3(b), the real part of the normal velocity in region II $\phi_{2z}(r,z)$ matches well with the symmetric normal velocity in the porous region III $\phi_{3z}(r,z)$ at *z* = −*L*. A comparable trend is observed in the anti-symmetric case shown in Fig 4. Specifically, Fig 4(a) demonstrates that the real part of the anti-symmetric membrane displacement *U*_1_ closely matches the corresponding normal velocity $\phi_{1z}(r,z)$ at *z* = −2*L*. In Fig 4(b), the anti-symmetric normal velocity in region II $\phi_{2z}(r,z)$ aligns well with the anti-symmetric normal velocity in the porous region III $\phi_{3z}(r,z)$ at *z* = −*L*. These results confirm the proper satisfaction of matching conditions at the interfaces for both symmetric and anti-symmetric configurations.

**Fig 5:**
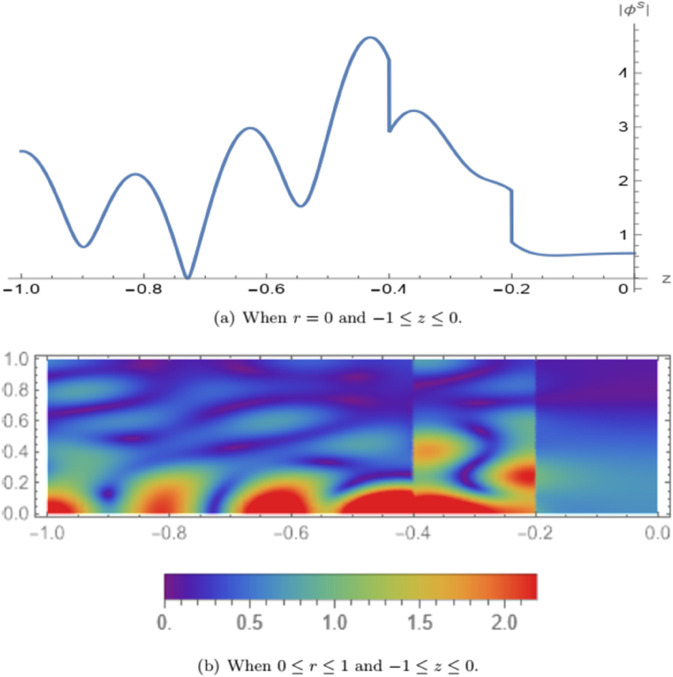
For symmetric case: The absolute value of the fluid potential $\left|\phi^{s}\right|$.

**Fig 6:**
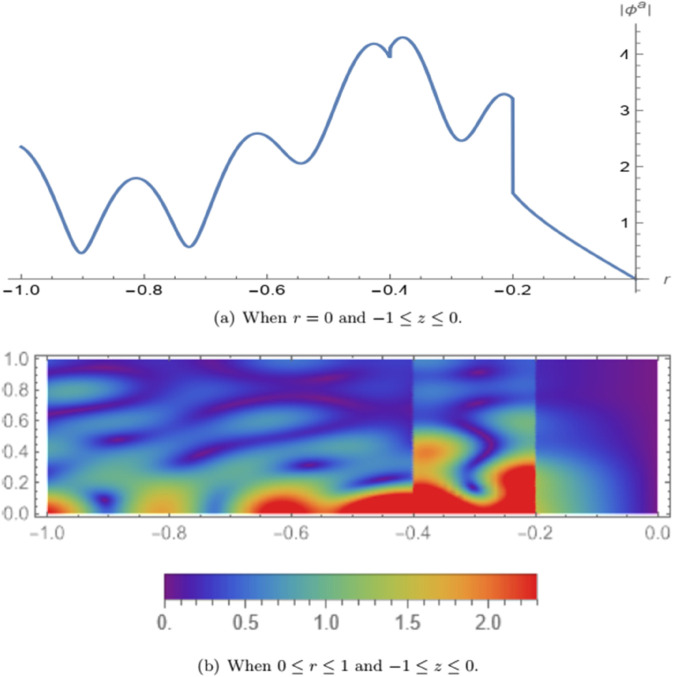
For anti-symmetric case: The absolute value of the fluid potential $\left|\phi^{a}\right|$.

In Fig 5, the absolute value of the symmetric fluid potential $\left|\phi^{s}\right|$ is illustrated. Fig 5(a) shows the variation of the symmetric potential along the axial coordinate *z* in the domain −1≤z≤0 with *L* = 0.2 and fixed radial position *r* = 0. In Fig 5(b), a three-dimensional view of $\left|\phi^{s}\right|$ is presented over the radial domain 0≤r≤1 and −1≤z≤0. It is observed that the wave mode traverses the membrane located at the interface *z* = −*L*, propagates through the air and porous regions, and reflects back upon encountering the rigid wall at *z* = 0. A significant portion of the incident wave energy is filtered within the air cavity, demonstrating the system’s attenuation capability. In Fig 6, the absolute value of the anti-symmetric fluid potential $\left|\phi^{a}\right|$ is depicted. Fig 6(a) presents the axial distribution of the anti-symmetric potential in the same domain −1≤z≤0 with *L* = 0.2 and *r* = 0. Fig 6(b) provides a three-dimensional plot of $\left|\phi^{a}\right|$ for 0≤r≤1. The results indicate that the mode crosses the membrane at *z* = −*L*, continues through the air and porous regions, and vanishes upon reaching the soft boundary at *z* = 0.

**Fig 7:**
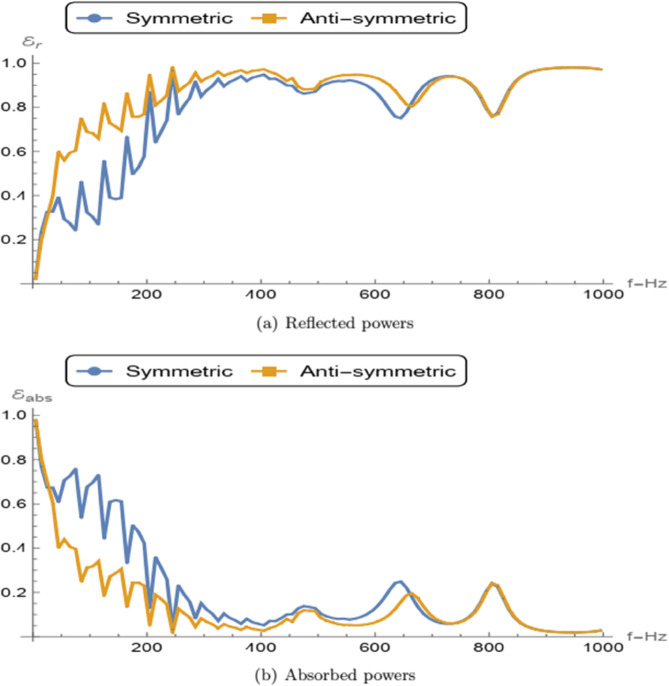
Comparison of the reflected and absorbed powers against frequency for symmetric and anti-symmetric problems.

The reflected and absorbed powers can be calculated by following the definition given in [25]. The expressions for reflected power for symmetric or anti-symmetric power when the incident field is scaled at unity can be expressed as

$${ℰ}_{r}=\frac{1}{ka^{2}}\sum_{n=0}^{𝒦}|A_{n}^{s\text{ or }a}{|}^{2}\eta_{n}R_{n},$$ (52)

where 𝒦 denotes the number of cut-on modes. The absorbed power due to the presence of the porous material is defined as ${ℰ}_{\text{abs}}=1-{ℰ}_{r}$, where ${ℰ}_{r}$ denotes the reflected power. In Fig 7, the reflected and absorbed powers are plotted as functions of frequency for both symmetric and anti-symmetric cases. From Fig 7(a), it is observed that the reflected power increases with frequency for both configurations. However, in the low-frequency regime, the anti-symmetric configuration exhibits higher reflection compared to the symmetric one. This behavior can be attributed to the boundary conditions: in the anti-symmetric case, the membrane is backed by an air–porous cavity terminated by a soft wall at *z* = 0, which reflects velocity modes back into the channel. Conversely, in the symmetric case, the rigid wall at *z* = 0 enforces a zero-velocity boundary condition, suppressing the reflected velocity modes. Fig 7(b) shows that the absorbed power decreases with increasing frequency in both cases. Notably, in the low-frequency range, the symmetric configuration achieves greater absorption than the anti-symmetric one. This is due to the fact that in the symmetric setting, the rigid wall at *z* = 0 facilitates the absorption of pressure modes propagating toward the backing channel. In contrast, for the anti-symmetric case, the soft wall at *z* = 0 imposes a zero-pressure condition, thereby limiting the pressure mode absorption in the porous layer.

By combining the symmetric and anti-symmetric problems, a multi-cavity waveguide structure can be constructed. The central porous cavity of length 2*L*, occupying the region –*L* < *z* < *L*, is flanked by two side cavities filled with air in the regions –2*L* < *z* < –*L* and *L* < *z* < 2*L*. Two membrane discs are positioned at *z* = −2*L* and *z* = 2*L*. The region *z* < –2*L* serves as the inlet, while *z* > 2*L* acts as the outlet. The fluid potentials in each region are defined as follows:

- $\phi_{1}(r,z)$ for *z* < –2*L*, 0 < *r* < *a* (inlet region),
- $\phi_{2}(r,z)$ for –2*L* < *z* < –*L*, 0 < *r* < *a* (left air cavity),
- $\phi_{3}(r,z)$ for –*L* < *z* < *L*, 0 < *r* < *a* (porous central cavity),
- $\phi_{4}(r,z)$ for *L* < *z* < 2*L*, 0 < *r* < *a* (right air cavity),
- $\phi_{5}(r,z)$ for *z* > 2*L*, 0 < *r* < *a* (outlet region).

The membrane displacements are denoted as:


$$U_{1}(r)\;\text{at}\;z=-2L,\;U_{2}(r)\;\text{at}\;z=2L,\;\text{for }0<r<a.$$

These fluid potentials and membrane displacements can be expressed in terms of the symmetric (*s*) and anti-symmetric (*a*) components as follows:


$$\phi_{1}(r,z)=\frac{1}{2}\left\{\phi_{1}^{s}(r,z)+\phi_{1}^{a}(r,z)\right\},\;\phi_{5}(r,z)=\frac{1}{2}\left\{\phi_{1}^{s}(r,z)-\phi_{1}^{a}(r,z)\right\},$$

$$\phi_{2}(r,z)=\frac{1}{2}\left\{\phi_{2}^{s}(r,z)+\phi_{2}^{a}(r,z)\right\},\;\phi_{4}(r,z)=\frac{1}{2}\left\{\phi_{2}^{s}(r,z)-\phi_{2}^{a}(r,z)\right\},$$

$$\phi_{3}(r,z)=\frac{1}{2}\left\{\phi_{3}^{s}(r,z)+\phi_{3}^{a}(r,z)\right\}+\frac{1}{2}\left\{\phi_{3}^{s}(r,z)-\phi_{3}^{a}(r,z)\right\},$$

$$U_{1}(r)=\frac{1}{2}\left\{U^{s}(r)+U^{a}(r)\right\},\;U_{2}(r)=\frac{1}{2}\left\{U^{s}(r)-U^{a}(r)\right\}.$$

**Fig 8:**
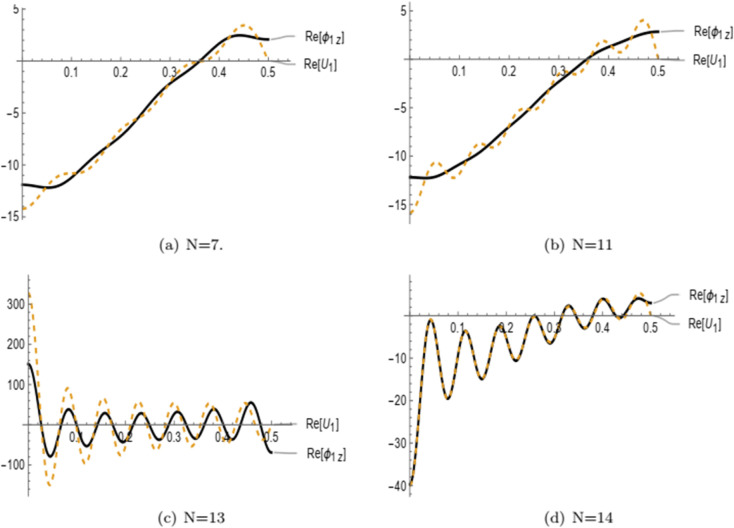
The real parts of normal velocity and displacement at z=−2L with different values of truncation parameter.

In Fig 8, the real parts of the normal velocity $\phi_{1z}(r,z)$ and displacement *U*_1_ are plotted as functions of the radial coordinate *r* at the interface *z* = −2*L*, where 0 < *r* < *a*, for different values of the truncation parameter N=7,11,13, and 14. As the number of retained modes increases, more wave modes are effectively cut-on and captured in the solution. The results demonstrate that the series solution exhibits point-wise convergence: the oscillatory features and modal interactions become more accurately resolved with higher truncation levels. In particular, the curves show significantly improved agreement at *N* = 14, indicating a better satisfaction of the matching conditions at the interface. This underscores the importance of selecting a sufficiently large truncation parameter to achieve an accurate and stable numerical representation of the physical fields. A convergence study was carried out by increasing the truncation parameter *N*. It was observed that the results stabilize beyond *N* = 13, with negligible changes for higher values. Therefore, *N* = 15 was selected in all computations to ensure accurate and convergent results.

**Fig 9:**
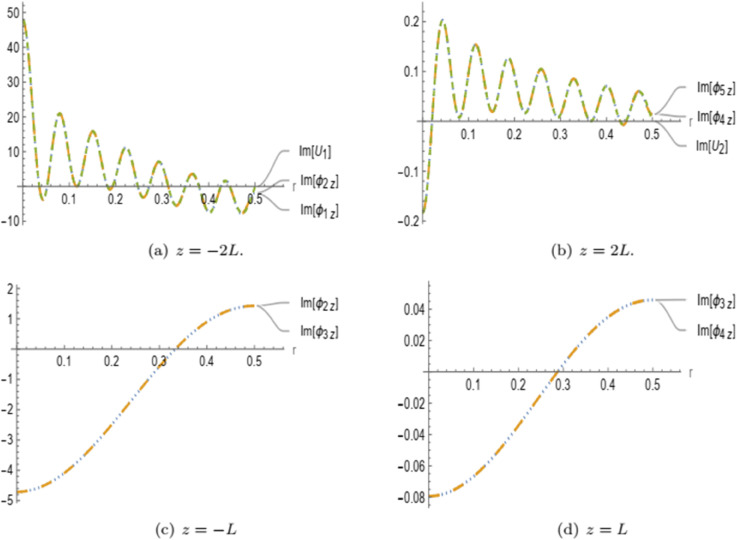
The imaginary parts of normal velocities and displacements are shown at the interfaces z=±L and ±2L.

Fig 9 presents the imaginary parts of the normal fluid velocities and membrane displacements at the interfaces z=±L and z=±2L. In Fig 9(a), it is observed that the normal velocities in the inlet region and the left air cavity, along with the membrane displacement, coincide at *z* = −2*L*, confirming the matching conditions imposed for both symmetric and anti-symmetric formulations. Similarly, Fig 9(b) illustrates the matching at the opposite boundary *z* = 2*L*, where the normal velocities in the right air cavity, the outlet region, and the membrane displacement align precisely. Further, Fig 9(c) shows that the normal velocities in the left air cavity and the central porous cavity match at *z* = −*L*, consistent with the prescribed continuity conditions. This behavior is mirrored on the opposite side in Fig 9(d), where the normal velocities of the central cavity and the right air cavity also match at *z* = *L*. The real parts of the velocities, exhibiting identical matching behavior, are not presented here to avoid redundancy, as their graphical representation is qualitatively similar.

The reflected, transmitted, and absorbed powers are computed using the definitions provided in [25]. The reflected power is given by

$${ℰ}_{r}=\frac{1}{ka^{2}}\sum_{n=0}^{𝒦}|A_{n}{|}^{2}\eta_{n}R_{n},$$ (53)

while the transmitted power is expressed as

$${ℰ}_{t}=\frac{1}{ka^{2}}\sum_{n=0}^{𝒦}|H_{n}{|}^{2}\eta_{n}R_{n}.$$ (54)

Here, the coefficients $A_{n}=A_{n}^{s}+A_{n}^{a}$ and $H_{n}=A_{n}^{s}-A_{n}^{a}$ are obtained from the symmetric and anti-symmetric components of the solution, and 𝒦 denotes the number of propagating (cut-on) modes. The absorbed power, accounting for energy loss due to the porous material, is defined as


$${ℰ}_{\text{abs}}=1-{ℰ}_{r}-{ℰ}_{t},$$

where ${ℰ}_{r}$ and ${ℰ}_{t}$ represent the reflected and transmitted powers, respectively.

**Fig 10:**
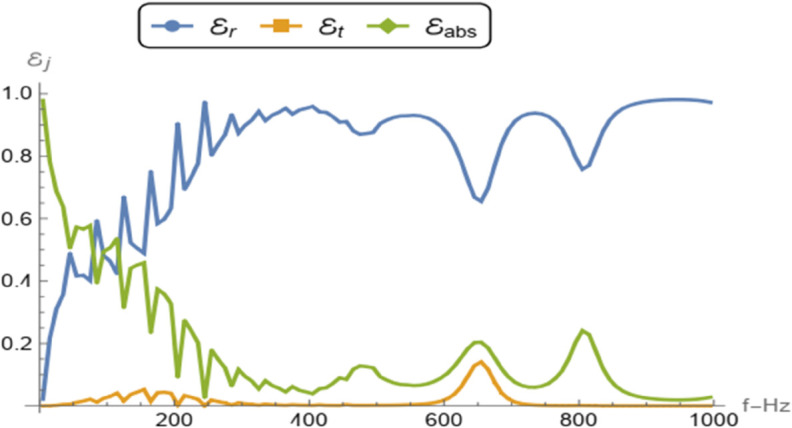
The reflected, transmitted, and absorbed powers are shown against frequency.

**Fig 11:**
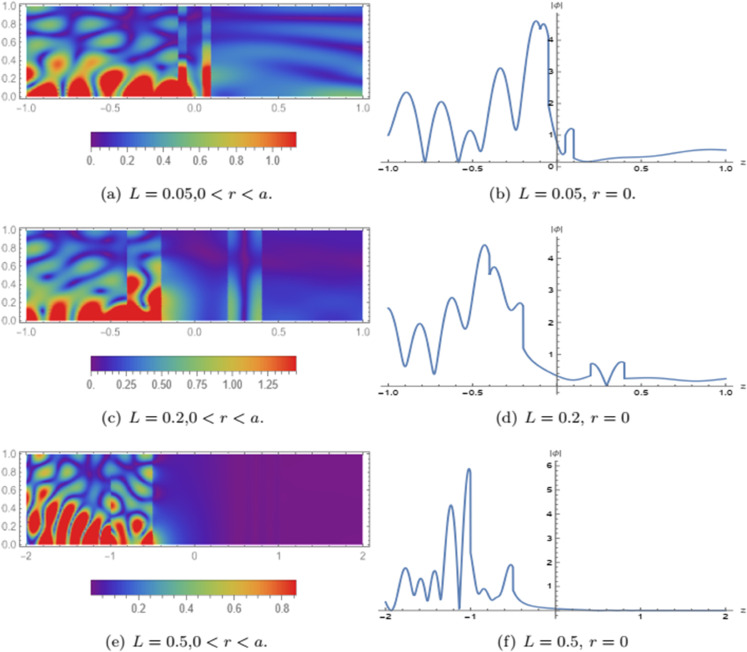
The impact of changing length L on the acoustics mode filtering when –2 < z < 2, a=0.1.

Fig 10 presents the frequency-dependent behavior of the reflected (${ℰ}_{r}$), transmitted (${ℰ}_{t}$), and absorbed (${ℰ}_{\text{abs}}$) powers. The duct radius is taken as *a* = 0.1 and the cavity length as *L* = 0.3, with the modal expansions truncated at *N* = 15 terms. As observed in the figure, the reflected power dominates across the entire frequency range. It exhibits an increasing trend with frequency and shows oscillatory patterns at higher frequencies, which reflect the effects of wave interference and structural resonance. This indicates a strong impedance mismatch leading to significant wave reflection due to the combined influence of the membrane discs and internal cavity configuration. The absorbed power ${ℰ}_{\text{abs}}$ is prominent in the low-frequency regime, highlighting the effectiveness of the porous medium in attenuating low-frequency waves. However, as frequency increases, absorption decreases gradually, with a few distinct peaks at intermediate frequencies that suggest resonance phenomena between the acoustic waves and the cavity structure. In contrast, the transmitted power ${ℰ}_{t}$ remains very small across the entire frequency range, indicating that only a negligible portion of the incident energy propagates through the system. This minimal transmission is primarily due to the presence of the membrane discs at the boundaries z=±2L and the air cavities within the structure, both of which act as strong barriers to wave propagation. Overall, the figure demonstrates the dominant role of reflection and frequency-selective absorption in the system, confirming its potential effectiveness in applications such as sound insulation, noise suppression, or frequency filtering where high reflection and controlled energy dissipation are desired. Fig 11 illustrates the influence of varying the cavity length *L* on acoustic wave mode filtering, within the domain –2 < *z* < 2 and fixed duct radius *a* = 0.1. The left column displays the spatial distribution of the real part of the fluid potential in the *z*-*r* plane, while the right column shows the corresponding axial profiles along the duct centerline at *r* = 0.

In subfigures (a) and (b), the cavity length is *L* = 0.05, representing a relatively short porous segment. The wave propagation shows partial penetration into the porous cavity with visible interference patterns and relatively strong transmitted components. The centerline plot (b) confirms this observation by exhibiting non-negligible wave amplitudes beyond the cavity, indicating limited filtering performance for such a small length. Increasing the cavity length to *L* = 0.2, as shown in subfigures (c) and (d), significantly enhances the attenuation of the wave field within the porous region. The spatial field in (c) reveals reduced transmission, and the wavefronts decay more noticeably inside the central region. The corresponding centerline profile in (d) shows a steeper drop in amplitude, highlighting improved energy dissipation and reflection due to the longer interaction path within the cavity.

When the cavity length is further increased to *L* = 0.5, depicted in subfigures (e) and (f), the porous layer acts as a highly effective filter. The wave penetration into the cavity is strongly suppressed, and nearly all incident energy is either reflected or absorbed. The centerline plot in (f) confirms a sharp decay in wave amplitude near the cavity entrance, with negligible transmission beyond the porous segment. These results clearly demonstrate that increasing the length of the porous region enhances the filtering efficiency by enabling more interaction between the acoustic field and the dissipative material, thereby improving both reflection and absorption capabilities.

To facilitate replication, we summarize a ready-to-build configuration that realizes the proposed mode filter. *Geometry:* straight circular duct of radius a=0.10m with a centrally lined segment of length 2*L*; interfaces at z=±L carry clamped circular membrane discs. *Parameters:* air $\left(\rho =1.2043\;kg/m^{3},\;c=343.5\;m/s\right)$; membrane surface density $\rho_{m}=0.1715\;kg/m^{2}$ and tension T=350N/m; porous insert modeled by a Delany–Bazley impedance with standard coefficients for a fibrous liner (flow resistivity as stated in the numerical data). *Solver/verification:* the mode-matching/Galerkin scheme is truncated at *N* = 15 (unless otherwise noted), and we report (i) interface matching (fluid normal velocity equals membrane velocity at z=±L), (ii) energy balance *E*_*r*_  +  *E*_*t*_  +  $E_{abs}=1$ computed from the solved amplitudes, and (iii) truncation convergence with respect to *N*. Frequency-scan results document selective reflection and absorption (low *E*_*t*_ on passbands), i.e., practical mode filtering. Increasing *L* strengthens attenuation and narrows the transmitted band, providing a simple tuning knob.

## 6 Summary and conclusion

This study presents an analytical investigation of acoustic wave propagation and mode filtering in a cylindrical waveguide system with flexible membranes and a centrally placed porous cavity. By employing a mode-matching formulation based on symmetric and anti-symmetric field decomposition, the coupled fluid-structure problem was solved efficiently for both reflection and transmission analyses. The configuration under consideration consists of a central porous cavity bounded by two air-filled side cavities and terminated by membrane discs. The reflected, transmitted, and absorbed powers were calculated by combining the symmetric and anti-symmetric potentials. Results demonstrate that the structure predominantly reflects incoming wave energy, with the reflected power increasing with frequency, while the transmitted power remains minimal throughout. The absorbed power is significant at low to intermediate frequencies, indicating efficient damping by the porous core, although this effect diminishes at higher frequencies. Field plots and axial profiles were used to examine the effect of varying the porous cavity length *L*. It was observed that increasing *L* enhances acoustic attenuation by enabling more effective energy dissipation within the porous region. Shorter lengths allow partial transmission and weaker filtering, while longer cavities result in strong suppression of wave propagation, confirming the role of cavity geometry in controlling acoustic energy distribution. Overall, the study confirms that the proposed multilayered configuration acts as an efficient frequency-dependent acoustic filter. The combination of membranes, air gaps, and porous media provides a tunable framework for noise reduction and wave manipulation. The analytical model developed here offers physical insight into wave interaction with dissipative structures and can serve as a foundation for designing compact acoustic filters and silencers in practical applications. It should be noted that the present model is based on several simplifying assumptions. For instance, mean flow effects and three-dimensional non-axisymmetric disturbances are not included in the current formulation. Similarly, the porous material is modeled using empirical relations (Delany–Bazley type), which may lose accuracy at very low frequencies or for materials with complex microstructures. Moreover, only linear fluid–structure interactions are considered, whereas nonlinear effects may become relevant at higher amplitudes. These limitations suggest directions for future research, including the incorporation of flow effects, refined porous models, and experimental validation to complement the analytical framework.

Open questions and promising directions for future work include: (i) quantifying the influence of mean flow on the coupled membrane–porous response and extending mode-matching treatments to include flow-advection and shear layers; (ii) assessing the limits of empirical porous models and integrating microstructure-aware or bi-phasic porous formulations for improved broadband accuracy; (iii) developing analytic/semi-analytic approaches to include non-axisymmetric and weakly nonlinear membrane dynamics to capture higher-amplitude behavior; and (iv) performing controlled experimental validation and uncertainty quantification to benchmark analytical predictions. Addressing these questions will broaden the practical relevance of analytic mode-matching frameworks and enable closer coupling with design and control strategies.
